# miR-624-5p promoted tumorigenesis and metastasis by suppressing hippo signaling through targeting PTPRB in osteosarcoma cells

**DOI:** 10.1186/s13046-019-1491-6

**Published:** 2019-12-11

**Authors:** Yongjun Luo, Wei Liu, Pengyu Tang, Dongdong Jiang, Changjiang Gu, Yumin Huang, Fangyi Gong, Yuluo Rong, Dingfei Qian, Jian Chen, Zheng Zhou, Shujie Zhao, Jiaxing Wang, Tao Xu, Yongzhong Wei, Guoyong Yin, Jin Fan, Weihua Cai

**Affiliations:** Department of Orthopaedics, the First Affiliated Hospital of Nanjing Medical University, Nanjing, 210029 Jiangsu China

**Keywords:** Osteosarcoma, miR-624-5p, PTPRB, Hippo, Metastasis

## Abstract

**Background:**

Accumulating evidence indicates that aberrant microRNA (miRNA) expression contributes to osteosarcoma progression. This study aimed to elucidate the association between miR-624-5p expression and osteosarcoma (OS) development and to investigate its underlying mechanism.

**Methods:**

We analyzed GSE65071 from the GEO database and found miR-624-5p was the most upregulated miRNA. The expression of miR-624-5p and its specific target gene were determined in human OS specimens and cell lines by RT-PCR and western blot. The effects of miR-624-5p depletion or ectopic expression on OS proliferation, migration and invasion were evaluated in vitro using CCK-8 proliferation assay, colony formation assay, transwell assay, would-healing assay and 3D spheroid BME cell invasion assay respectively. We investigated in vivo effects of miR-624-5p using a mouse tumorigenicity model. Besides, luciferase reporter assays were employed to identify interactions between miR-624-5p and its specific target gene.

**Results:**

miR-624-5p expression was upregulated in OS cells and tissues, and overexpressing miR-624-5p led to a higher malignant level of OS, including cell proliferation, migration and invasion in vitro and in vivo. Protein tyrosine phosphatase receptor type B (PTPRB) was negatively correlated with miR-624-5p expression in OS tissues. Using the luciferase reporter assay and Western blotting, PTPRB was confirmed as a downstream target of miR-624-5p. PTPRB restored the effects of miR-624-5p on OS migration and invasion. The Hippo signaling pathway was identified as being involved in the miR-624-5p/PTPRB axis.

**Conclusions:**

In conclusion, our results suggest that miR-624-5p is a negative regulator of PTPRB and a risk factor for tumor metastasis in OS progression.

## Background

Osteosarcoma (OS) is the most commonly seen malignant bone tumor worldwide (accounting for nearly 60% of bone malignancies) and severely affects the daily lives of patients [1, 2]. It typically occurs among adolescents and young adults and has been considered to be a consequence of malignant mesenchymal cell differentiation [3, 4]. Lung metastasis is found among 10–25% of patients and death from pulmonary metastasis can happen within one year [5]. Although great effort has been made to treat and cure OS, patients with metastatic or recurrent OS still have a low survival rate. Therefore, developing more potent therapeutic strategies for OS is sorely needed.

MicroRNAs (miRNAs) are small non-coding endogenous RNAs and consist of approximately 18 to 24 nucleotides [6]. They negatively regulate target genes by combining with the 3′-untranslated regions (3′-UTRs) of the target mRNAs and degrading them [7]. MicroRNAs are considered to participate in the development of various tumors, including osteosarcoma, and regulate cell proliferation, apoptosis, and tumorigenesis through multiple signaling pathways [8–15].

Lower levels of miR-624-5p have been detected in hepatoblastoma tissues compared to normal livers. In addition, miR-624-5p induces cell senescence in vitro and blocks tumor growth in vivo by directly targeting β-catenin 3′-UTRs [16]. miR-624 was reported to serve not only as prognostic biomarkers for cancer treatment outcome but also as interventional agents to modulate desired chemosensitivity [17]. Another review has shown that miR-624 is commonly downregulated in recurrent prostate cancer samples [18]. Downstream genes of miR-624-5p (NRP1, BIRC5, ABCB1, SIX1, CCND1, and FGF9) have been suggested to be involved in cell survival, proliferation, migration, cell cycle progression, tumor growth, and drug resistance [16]. However, to the best of our knowledge, the exact roles of miR-624-5p in osteosarcoma growth and pulmonary metastasis are still unclear. In this study, we investigated the roles of miR-624-5p in OS and demonstrated that miR-624-5p is highly expressed in OS cell lines and tumors. By experiments both in vivo and in vitro, we successfully suppressed osteosarcoma cells growth and metastasis by downregulating miR-624-5p.

The Hippo signaling pathway has been confirmed to have an inhibitory role in the regulation of tumorigenesis in various tissues [19–21]. Hippo successively activates LATS kinases and phosphorylates YAP, leading to the cytoplasmic retention of YAP [22]. Protein tyrosine phosphatase receptor type B (PTPRB) is known as a type of vascular endothelial protein tyrosine phosphatase (VE-PTP) and is a potential target of miR-624-5p (predicted by TargetScan). Additionally, PTPRB is known to participate in the formation, maintenance, and remodeling of blood vessels [23, 24]. Recent studies have shown that PTPRB may exert an effect on carcinogenesis and cancer development [25, 26]. However, the relationship between the miR-624-5p–PTPRB axis and the Hippo signaling pathway involved in OS proliferation, migration, and metastasis still demands further research.

## Methods

### Tissue samples and cell lines

This study was approved by the ethics committee of the First Affiliated Hospital of Nanjing Medical University. All human osteosarcomas and their adjacent normal muscle tissues were obtained from a total of 50 patients during biopsies in the Department of Orthopedics. Patients did not receive anticancer treatments like chemotherapy or radiotherapy before all the tissue samples were obtained by biopsies. The tissues were collected and then immediately frozen in liquid nitrogen. The clinicopathological and demographic information of the patients is described in Table 1.

**Table 1:** Expression of miR-624-5p and PTPRB according to patients’ clinical features

		miR-624-5p expression			PTPRB expression		
Characteristics	Number	High group	Low group	*P* value	High group	Low group	P value
Age(y)							
< 18	29	14	15	0.70	15	14	0.96
≥ 18	21	9	12		11	10	
Gender							
Female	23	13	10	0.39	12	11	0.61
Male	27	12	15		16	11	
Location							
Femur/Tibia	39	18	21	0.96	20	19	0.47
Elsewhere	11	5	6		7	4	
TNM stage							
I	23	11	12	0.028^a^	15	8	0.012^a^
II/III	27	21	6		8	19	
Tumor size (cm)							
< 5	28	12	16	0.014^a^	17	11	0.042^a^
≥ 5	22	17	5		7	15	
Lung metastasis							
Yes	21	18	3	0.012^a^	6	15	0.01^a^
No	29	15	14		19	10	

^a^P < 0.05(Chi-square test)

### Cell culture

The human OS cell lines including U2OS, MG63, SW1353, HOS, and Saos-2 and the normal human osteoblast cell line hFOB1.19 were obtained from the American Type Culture Collection (ATCC, Manassas, VA, USA). For each experiment, OS cells were sustained in Dulbecco’s modified Eagle’s medium (DMEM; Hyclone, UT, USA) supplemented with 10% fetal bovine serum (FBS; Gibco Laboratory, Grand Island, NY) and 1% penicillin/streptomycin (Gibco, Carlsbad, CA).

### Establishment of stably transfected cells

We purchased LV3-has-miR-624-5p-pre-microRNA vector (miR-624-5p mimics), LV3-has-miR-624-5p-sponge inhibitor vector (miR-624-5p inhibitor), vector containing the PTPRB DNA sequence (PTPRB), and lentiviral vector containing PTPRB siRNA sequence (siPTPRB) constructs from GenePharma (Shanghai, China). Osteosarcoma cells were infected with the lentiviruses and then selected using 7 μg/mL puromycin (Sigma-Aldrich, USA).

### Real-time quantitative polymerase chain reaction (PCR)

Following biopsies, tissues samples were stored at liquid nitrogen, and pulverized before the total RNA extraction. After being extracted from tissues and cells with Trizol (Invitrogen, USA), total RNA was resuspended in DEPC-treated H2O, and the concentration and purity were confirmed at 260 nm. Reverse transcription was performed using the PrimeScript RT Reagent Kit (Takara, China) according to the manufacturer’s protocol. SYBR Green Master (TaKaRa) was used for the quantitative PCR measure. The levels of U6 and GAPDH served as the internal control. The primers for PTPRB, miR-624-5p, U6, and GAPDH were purchased from RiboBio (Guangzhou, China). The sequences of the primers are as follows: PTPRB forward: 5′-ACAACACCACATACGGATGTAAC-3′; PTPRB reverse: 5′-CCTAGCAGGAGGTAAAGGATCT-3′; GAPDH forward: 5′-TAATCTTCGCCTTAATACTT-3′; GAPDH reverse 5′-AGCCTTCATACATCTCAA-3′; U6 forward: 5′-CTCGCTTCGGCAGCACA-3′; and U6 reverse: 5′-AACGCTTCACGAATTTGCGT-3′.

### Invasion assay

Transwell chambers (Millipore, USA) were used to measure cell invasion. Briefly, for invasion assays, cells were seeded on the upper surface of Matrigel-coated membrane inserts. After 24 h, cells that had invaded across the Transwell membrane were fixed with 4% paraformaldehyde and stained with 0.5% crystal violet for 30 min. The invasive cells were counted in three random microscopic views and photographed under an optical microscope (Nikon, Tokyo, Japan).

### Wound-healing assay

A wound-healing assay was performed to assess cell migration capability. OS cells were seeded in six-well plates and were grown to 80–90% confluence overnight. The cells were scratched using a sterile 200 μL pipette tip, and the wound recovery was observed after 0 and 24 h.

### 3D spheroid BME cell invasion assay

For 3D spheroid BME cell invasion assays, 20 μL cell suspension (1000 cells) was placed on the lid of a 10-cm-diameter dish. The lid was then inverted over dishes with 10 mL PBS. After culturing the hanging drops for two days, the cellular aggregates were obtained and implanted into 3D collagen I gels (PureCol, Inamed, Fremont, CA, USA), which were prepared by adjusting the pH to 7.5 with NaOH and DMEM and 2% FBS. After polymerization at 37 °C, the collagen I gel was overlaid with 300 μL of DMEM containing 10% FBS. After 48 h, the motion of the cells was monitored as fully formed under microscopy.

### Cell counting Kit-8 assay and colony formation assay

Transfected osteosarcoma cells were cultured in 96-well plates (2 × 10^3^ cells with 100 μL culture medium per well) and incubated for 24, 48, 72, 96, and 120 h. Cell proliferation was analyzed by the Cell Counting Kit-8 (CCK-8) (Dojindo, Japan) according to the manufacturer’s instructions. 10 μL of CCK8 solution in fresh culture medium was added every 24 h and incubated for 2 h at 37 °C, and the optical density (OD) value at 450 nm wavelength was determined using a microplate reader (ELx800, Bio-Tek, USA). For the colony formation assay, cells were cultured in Petri dishes with 10% FBS. The colonies were stained using crystal violet after 14 days and counted.

### Immunofluorescence analysis

Transfected cells were fixed with 4% paraformaldehyde and permeabilized with 0.3% Triton X-100. The cells were then subjected to immunofluorescence staining with the primary antibodies to Hippo signaling genes, such as YAP and TAZ (Cell Signaling Technology, USA), for 16 h. Afterward, cells were washed with PBS and incubated with fluorescence-labeled secondary antibodies for 30 min. Finally, images were acquired and analyzed by fluorescence microscopy (Carl Zeiss Microscopy GmbH, Jena, Germany).

### Luciferase reporter assay

Possible miR-624-5p binding sites were acquired from a miRNA database (targetscan.org). Wild-type PTPRB (WT-PTPRB-3′-UTR) and mutant PTPRB (MUT-PTPRB-3′-UTR) were synthesized by GenePharma (Shanghai, China). Cells overexpressing miR-624-5p or its control were transfected with WT-PTPRB-3′-UTR and MUT-PTPRB-3′-UTR. Cells were collected 48 h after transfection, and firefly luciferase activity was determined by the Dual-Luciferase Assay System (Promega, Madison, WI, USA). The results were normalized with Renilla luciferase.

### Western blotting

Proteins were extracted, and their concentrations were measured with the BCA protein assay kit (Beyotime, Shanghai, China). The proteins were then electrophoresed by 10% SDS-PAGE and transferred to PVDF membranes (Bio-Rad, Hercules, CA, USA). Next, the membranes were blocked in 5% bovine serum albumin (BSA) and incubated overnight at 4 °C with specific primary antibodies (1:1000). Rabbit anti-PTPRB (Abcam, Cambridge, UK), GAPDH, N-cadherin, E-cadherin, vimentin, p-LATS1, p-YAP, p-TAZ, LATS1, YAP, and TAZ (Cell Signaling Technology) antibodies were used. After that, the membranes were incubated with the secondary antibody (1:5000) at room temperature for 2 h. Reacting bands were visualized using ECL reagent (Thermo Fisher Scientific), and the density of protein bands was semi-quantified using ImageJ.

### Immunohistochemistry

All specimens were fixed in 4% paraformaldehyde and embedded in paraffin. Next, the paraffin was cut into 4-μm sections and incubated overnight with the primary antibody for PTPRB (Abcam, Cambridge, UK). The sections were then incubated with the secondary antibody for 1 h and stained using 3,3-diaminobenzidine solution for 3 min. We selected three fields to measure the percentage of positive tumors and staining intensities.

### Animal experiments

The animal studies were approved by the Institutional Animal Care and Use Committee of the First Affiliated Hospital of Nanjing Medical University. Nude mice used for tumor growth assays were purchased from the Animal Model Institute of Nanjing University (Nanjing, China). The nude mice were randomly divided into four groups (*n* = 5 per group). Stable cells labeled with firefly luciferase (2 × 10^6^ cells) in 100 μL PBS were subcutaneously injected into the nude mice. The progression of xenograft growth were imaged on day 35 using the IVIS200 imaging system (Caliper Life Sciences, Waltham, MA, USA).

### Statistical analysis

All experiments were performed in at least three times, and data are expressed as the mean ± standard deviation. The χ2 test was used to analyze the association of miR-624-5p expression level with clinicopathological features. Independent *t*-tests were used to compare differences between the two groups. A paired *t*-test was used to analyze miR-624-5p and PTPRB mRNA levels in tissue samples. One-way or two-way ANOVA with Bonferroni post hoc test was used for multivariate analysis. Correlations were determined by Pearson’s correlation analysis. Statistical analyses were performed using SPSS, v. 22.0 (SPSS Inc., Chicago, IL, USA). *P* < 0.05 was considered as statistically significant.

## Results

### miR-624-5p is upregulated in OS cell lines and tissues

**Fig. 1:**
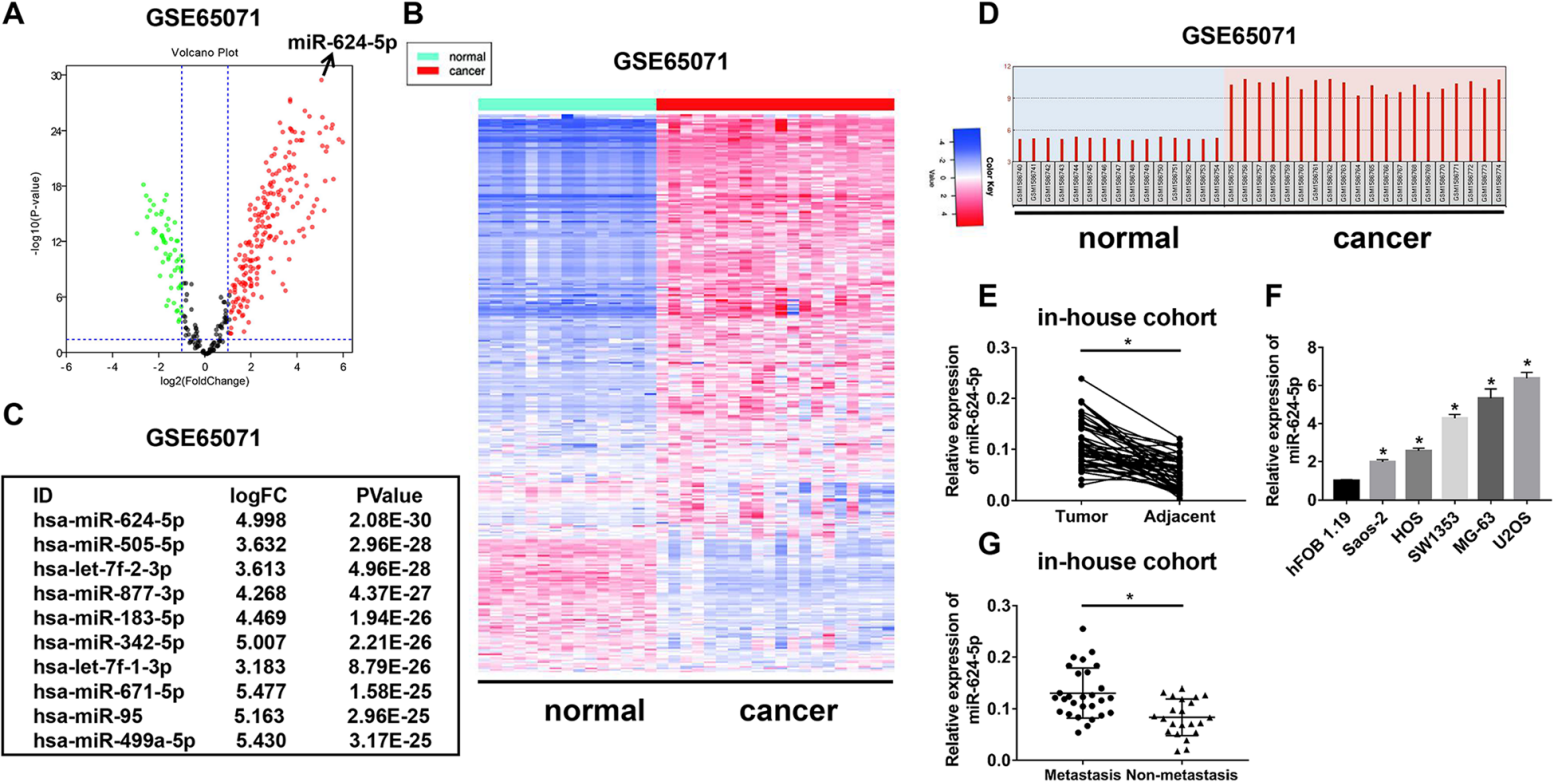
miR-624-5p is upregulated in osteosarcoma (OS) cell lines and tissues. **a.** The variation in miRNA expression between OS and normal tissues from GSE65071 was compared. **b.** A cluster heap map was used to present the up- and downregulated microRNAs (miRNAs) from GSE65071. **c.** The top 10 upregulated miRNAs are listed. **d–e.** miR-624-5p was significantly upregulated in OS tissues according to GSE65071 and in-house cohort. **f.** The relative expression of miR-624-5p was notably increased in OS cell lines (*n* = 4). **g.** miR-624-5p was highly expressed in patients with metastasis from in-house cohort

To determine the expression pattern of miRNAs in OS tissues, we analyzed GSE65071 from the GEO database based on the limma function. We compared the variation of miRNA expression between OS and normal tissues via volcano plots (Fig. 1a). In total, 204 miRNAs were upregulated in OS tissues with fold-changes greater than 2.0. Then, a cluster heap map was used to present the up- and downregulated miRNAs according to the miRNA expression levels (Fig. 1b). The top 10 upregulated miRNAs are listed in Fig. 1c. Among these, we found miR-624-5p was the most upregulated miRNA. Thus, we attempted to understand the mechanism of the tumor promoter role of miR-624-5p. According to the GEO database, miR-624-5p was significantly upregulated in OS tissue compared to normal tissue (Fig. 1d). Then, we performed real-time quantitative PCR (RT-qPCR) to examine miR-624-5p expression from 50 paired OS tissues and adjacent tissues to testify whether miR-624-5p was aberrantly expressed. Compared to peritumor samples, miR-624-5p expression was significantly increased in OS tissues (*P* = 0.000, Fig. 1e). Similarly, compared with miR-624-5p expression levels in the normal cell line hFOB 1.19, the relative expression of miR-624-5p was notably increased in OS cell lines, including Saos-2, HOS, SW1353, MG-63, and U2OS (*P* = 0.000, Fig. 1f). It was shown that miR-624-5p was highly expressed in patients with metastasis (*P* = 0.000, Fig. 1g). To better determine the clinical significance of miR-624-5p in OS, we defined the median expression level of miR-624-5p as a cutoff value and divided the patients into subgroups named low miR-624-5p and high miR-624-5p. As shown in Table 1, the expression level of miR-624-5p was significantly positively correlated with TNM stage, tumor size, and lung metastasis.

### Downregulating miR-624-5p inhibited OS cell invasion and migration in vitro

**Fig. 2:**
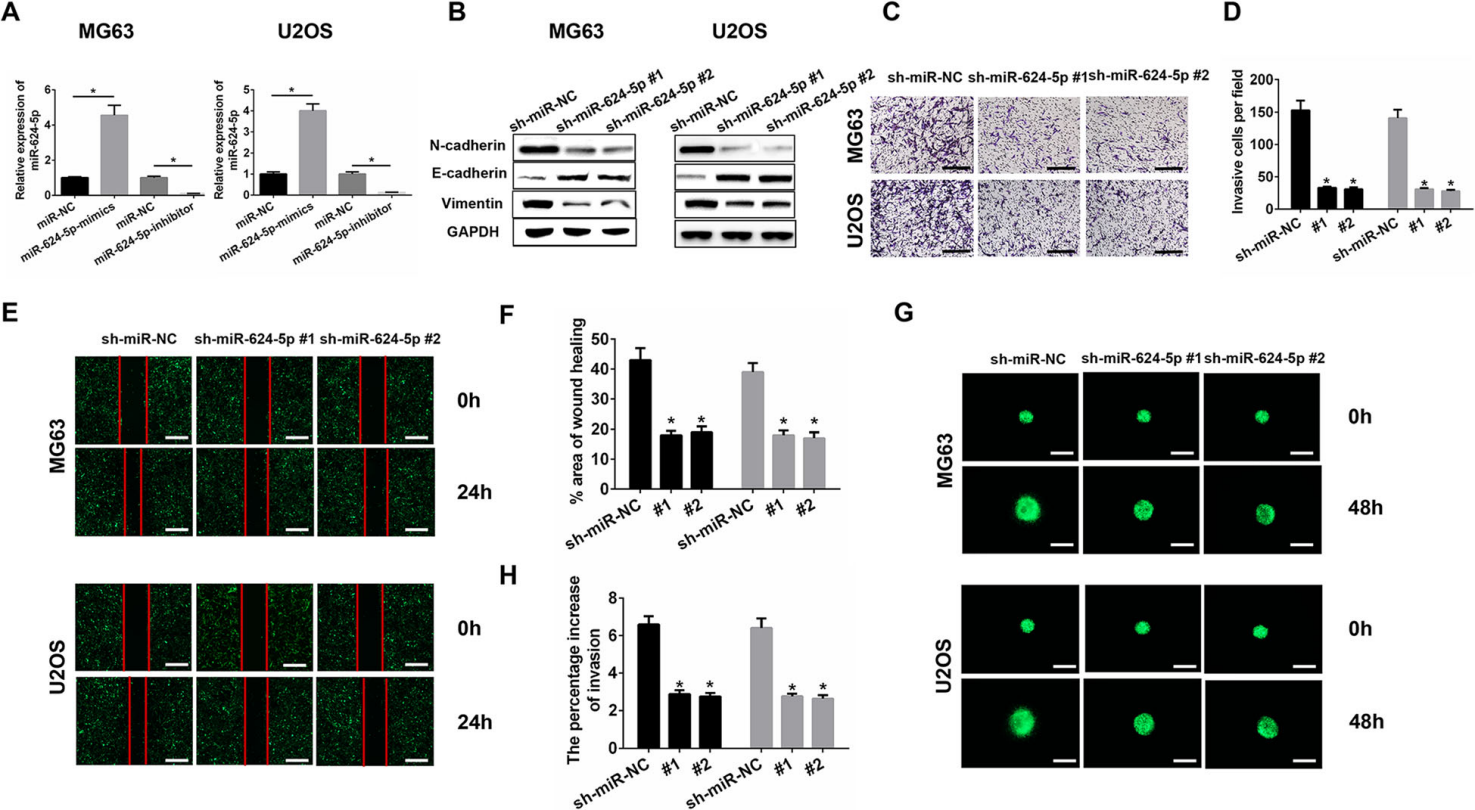
Downregulating miR-624-5p inhibited OS cell invasion and migration in vitro. **a.** miR-624-5p lentiviruses were successfully transfected into MG63 and U2OS cell lines (*n* = 3). **b.** sh-miR-624-5p #1 and sh-miR-624-5p #2 decreased the level of metastasis-related proteins in MG63 and U2OS (*n* = 3). **c–f.** The knockdown of miR-624-5p remarkably suppressed the invasion and migration of MG63 and U2OS cells (*n* = 4). **g–h.** 3D spheroid BME cell invasion assays confirm that downregulating miR-624-5p inhibited OS cell invasion (*n* = 4)

MG63 and U2OS were used for all the in vitro experiments. We confirmed the transfection efficiency of miR-624-5p lentiviruses using qRT-PCR. The results showed that miR-624-5p was significantly overexpressed in the mimics group and inhibited in the inhibitor group (MG63, *P* = 0.000; U2OS, *P* = 0.000; Fig. 2a). Western blot analysis showed that sh-miR-624-5p #1 and sh-miR-624-5p #2 increased E-cadherin levels and decreased the metastasis-related protein levels of N-cadherin and vimentin in MG63 and U2OS cells (Fig. 2b). The Transwell assay and wound-healing assay were performed to elucidate the effects of miR-624-5p on the invasion and migration of OS cells in vitro. The Transwell assay showed that the knockdown of miR-624-5p remarkably decreased the invasive cell number per field (MG63, *P* = 0.000; U2OS, *P* = 0.000; Fig. 2c, d). The wound-healing assay supported the results that inhibiting miR-624-5p could suppress the migration of MG63 and U2OS cells (MG63, *P* = 0.000; U2OS, *P* = 0.000; Fig. 2e, f). In 3D migration assays, MG63 and U2OS tumorspheres seeded in a 3D collagen matrix resembled the results observed in the Transwell and wound-healing assays (MG63, *P* = 0.000; U2OS, *P* = 0.000; Fig. 2g, h). Further, we evaluated the effect of miR-624-5p inhibition on OS cell proliferation by CCK8 and colony formation assays. As shown in Additional file 1: Figs. S1A and S1B, downregulating miR-624-5p showed no significant difference in the first three days, but significantly reduced cell proliferation after five days, indicating that miR-624-5p also has the potential to promote OS cell proliferation.

### miR-624-5p promotes OS cell invasion and migration in vitro

**Fig. 3:**
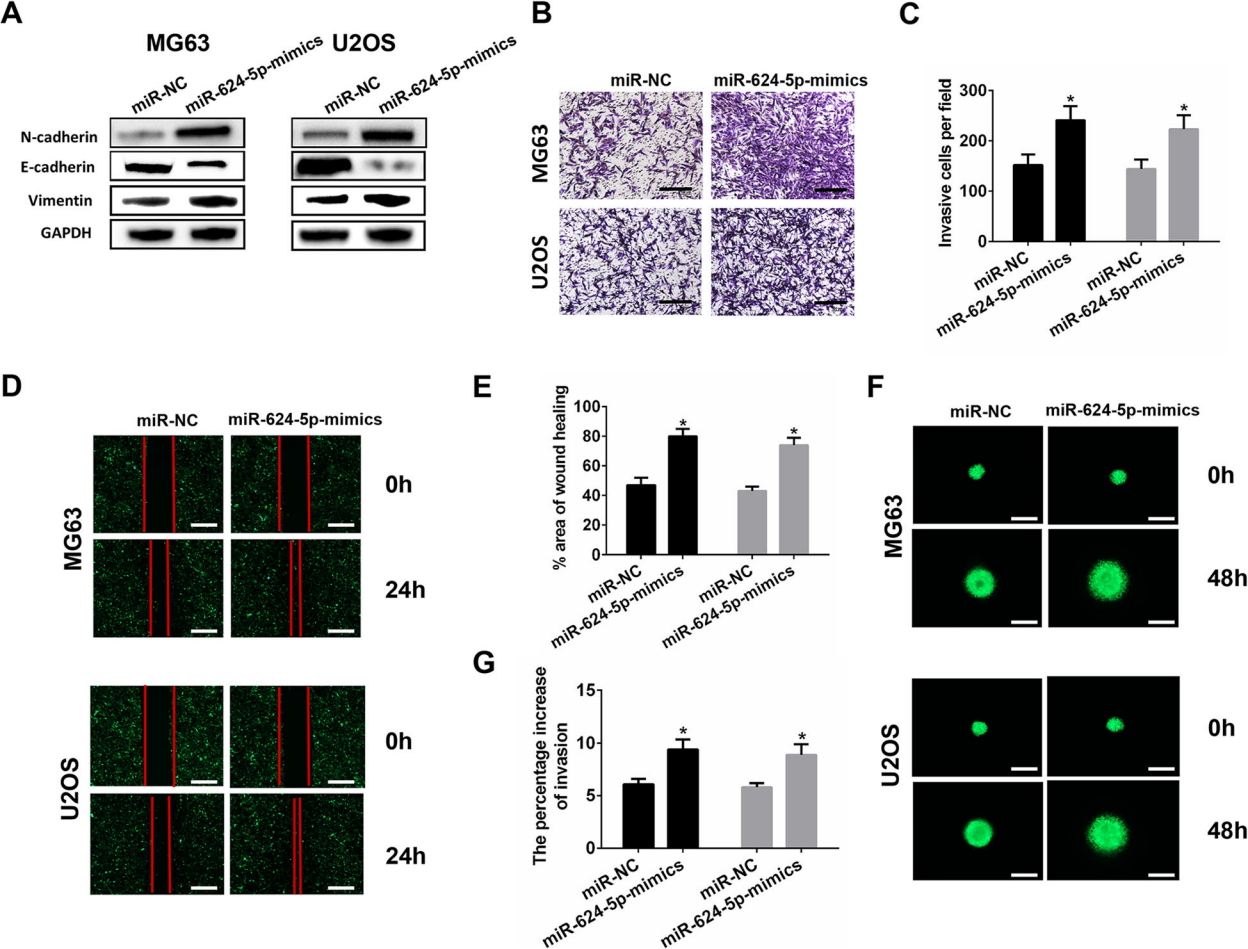
miR-624-5p promotes OS cell invasion and migration in vitro. **a.** Western blotting shows that miR-624-5p promoted the EMT progression (n = 3). **b–e.** miR-624-5p overexpression markedly accelerated cell invasion and migration in MG63 and U2OS cells (n = 4). **f–g.** MG63 and U2OS tumorspheres seeded in a 3D collagen matrix (n = 4)

Western blotting assay showed that E-cadherin expression was downregulated in the miR-624-5p mimics group, and N-cadherin and vimentin levels were upregulated in OS cells (Fig. 3a). For Transwell assays, miR-624-5p overexpression markedly promoted cell invasion in the MG63 and U2OS cells (MG63, *P* = 0.012; U2OS, *P* = 0.016; Fig. 3b, c), and the wound-healing assay showed consistent results with the Transwell assay results (MG63, *P* = 0.001; U2OS, *P* = 0.000; Fig. 3d, e). Further, we used 3D spheroid BME cell invasion assays to confirm the effects of miR-624-5p on invasion. The results were similar to those from the Transwell and wound-healing assays (MG63, *P* = 0.006; U2OS, *P* = 0.008; Fig. 3f, g). Furthermore, the CCK-8 assay and colony formation analysis revealed that upregulation of miR-624-5p significantly increased the proliferation of MG63 and U2OS cells (Additional file 1: Figs. S1C and S1D). These data collectively demonstrate that miR-624-5p mediates OS cell invasion and migration processes and proliferation.

### PTPRB is downregulated in OS tissues and is a target of miR-624-5p

**Fig. 4:**
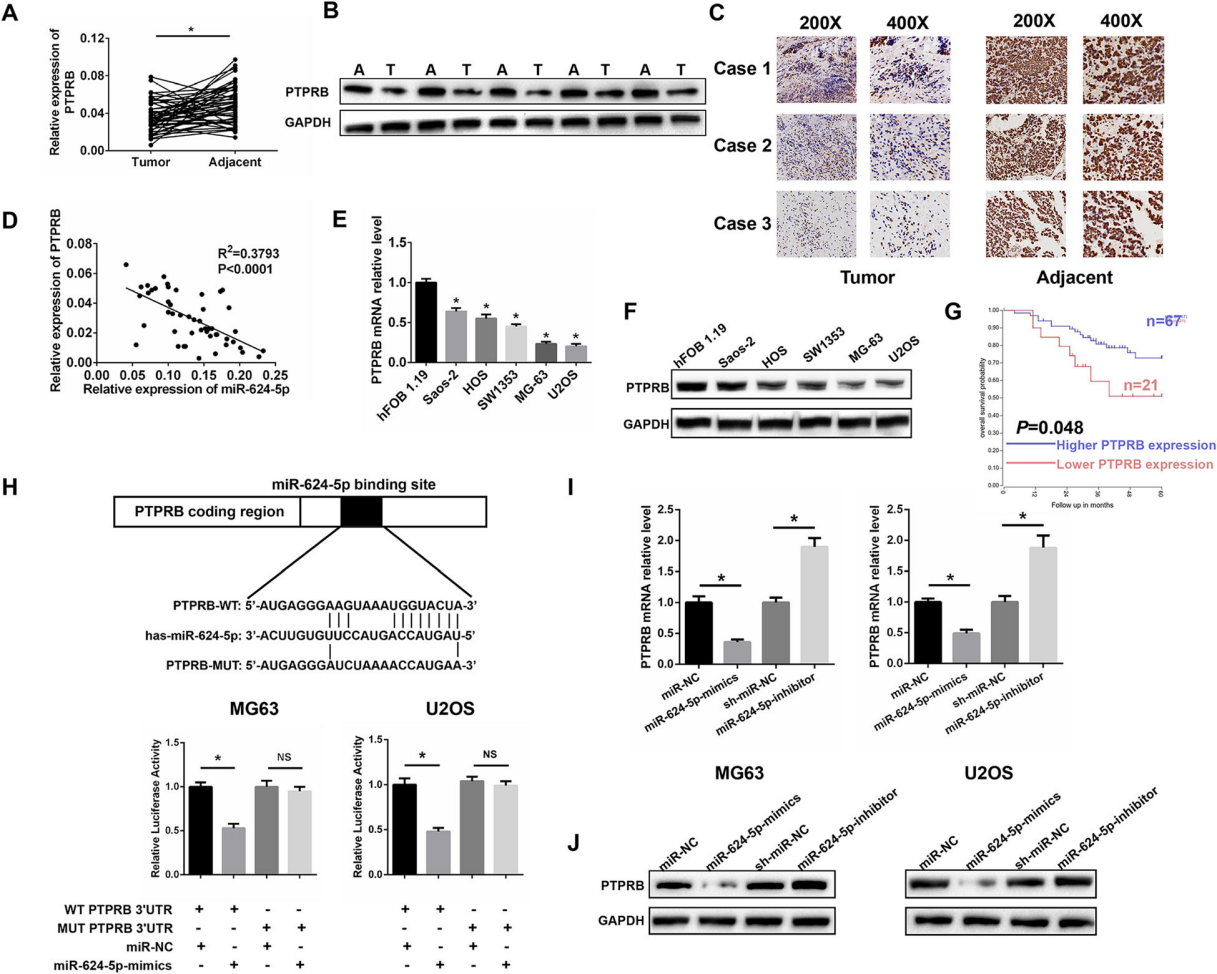
Protein tyrosine phosphatase receptor type B (PTPRB) is downregulated in OS tissues and is a target of miR-624-5p. **a–c.** PTPRB expression was markedly lower in OS tissues. **d.** PTPRB expression level was negatively related to miR-624-5p level in OS tissues. **e.** The mRNA levels of PTPRB were downregulated in several OS cell lines, especially in MG63 and U2OS cells (n = 4). **f.** MG63 and U2OS cells contained the least PTPRB protein compared with the other cell lines (n = 4). **g.** Kaplan-Meier analysis demonstrates that patients with high PTPRB expression levels had a much better prognosis based on an online database (https://hgserver1.amc.nl/cgi-bin/r2/main.cgi). **h.** The WT-PTPRB-3′-UTR and MUT-PTPRB-3′-UTR were synthesized. Overexpressed miR-624-5p markedly suppressed the luciferase activity of WT-PTPRB-3′-UTR but had no effect on MUT-PTPRB-3′-UTR in MG63 and U2OS cells (*n* = 5). **I.** qRT-PCR shows that the PTPRB mRNA expression level was negatively regulated by miR-624-5p (n = 4). **j.** Western blotting results support that miR-624-5p negatively controls the expression level of PTPRB (n = 3)

We analyzed the potential miR-624-5p targets using TargetScan. Among the candidate genes, we were particularly interested in PTPRB due to its potential tumor-suppressing role in carcinogenesis and cancer development. We performed qRT-PCR and Western blotting to investigate PTPRB expression in 50 paired OS tissues and adjacent tissues. PTPRB expression was markedly lower in OS tissues than the adjacent normal tissues (*P* = 0.001, Fig. 4a, b). Immunohistochemistry assays supported the above results (Fig. 4c). We demonstrated that the PTPRB expression level was negatively related to miR-624-5p in OS tissues with an R^2^ of 0.3793 (Fig. 4d). What is more, the mRNA levels of PTPRB were observed to be downregulated in several OS cell lines, especially in MG63 and U2OS cells (P = 0.001, Fig. 4e). Western blotting results further confirmed that MG63 and U2OS cells contained the least amount of PTPRB protein compared with the other cell lines (Fig. 4f). Also, we defined the median expression level of PTPRB as a cutoff value and divided the patients into subgroups named low PTPRB and high PTPRB. As demonstrated in Table 1, we found that the expression level of PTPRB was negatively correlated with TNM stage, tumor size, and lung metastasis. As shown in Fig. 4g, Kaplan-Meier analysis demonstrated that patients with a high level of PTPRB expression had a much better prognosis than those with weak expression (*P* = 0.048). Luciferase reporter assays were performed and showed that miR-624-5p could directly target PTPRB. WT-PTPRB-3′-UTR and MUT-PTPRB-3′-UTR were synthesized as shown in Fig. 4h. The overexpression of miR-624-5p markedly suppressed the luciferase activity of WT-PTPRB-3′-UTR but had no effect on MUT-PTPRB-3′-UTR in MG63 and U2OS cells (MG63, *P* = 0.004; U2OS, *P* = 0.003; Fig. 4h). qRT-PCR showed low PTPRB mRNA expression in cells transfected with miR-624-5p mimics; in contrast, a significantly high PTPRB expression was detected in cells with miR-624-5p inhibitor (MG63, *P* = 0.000; U2OS, *P* = 0.002; Fig. 4i). The Western blotting results supported that miR-624-5p negatively regulated the expression level of PTPRB in vitro (Fig. 4j). All of the data indicate that PTPRB is a direct target of miR-624-5p.

### PTPRB silencing abolishes the effects of miR-624-5p inhibitor on OS migration and invasion

**Fig. 5:**
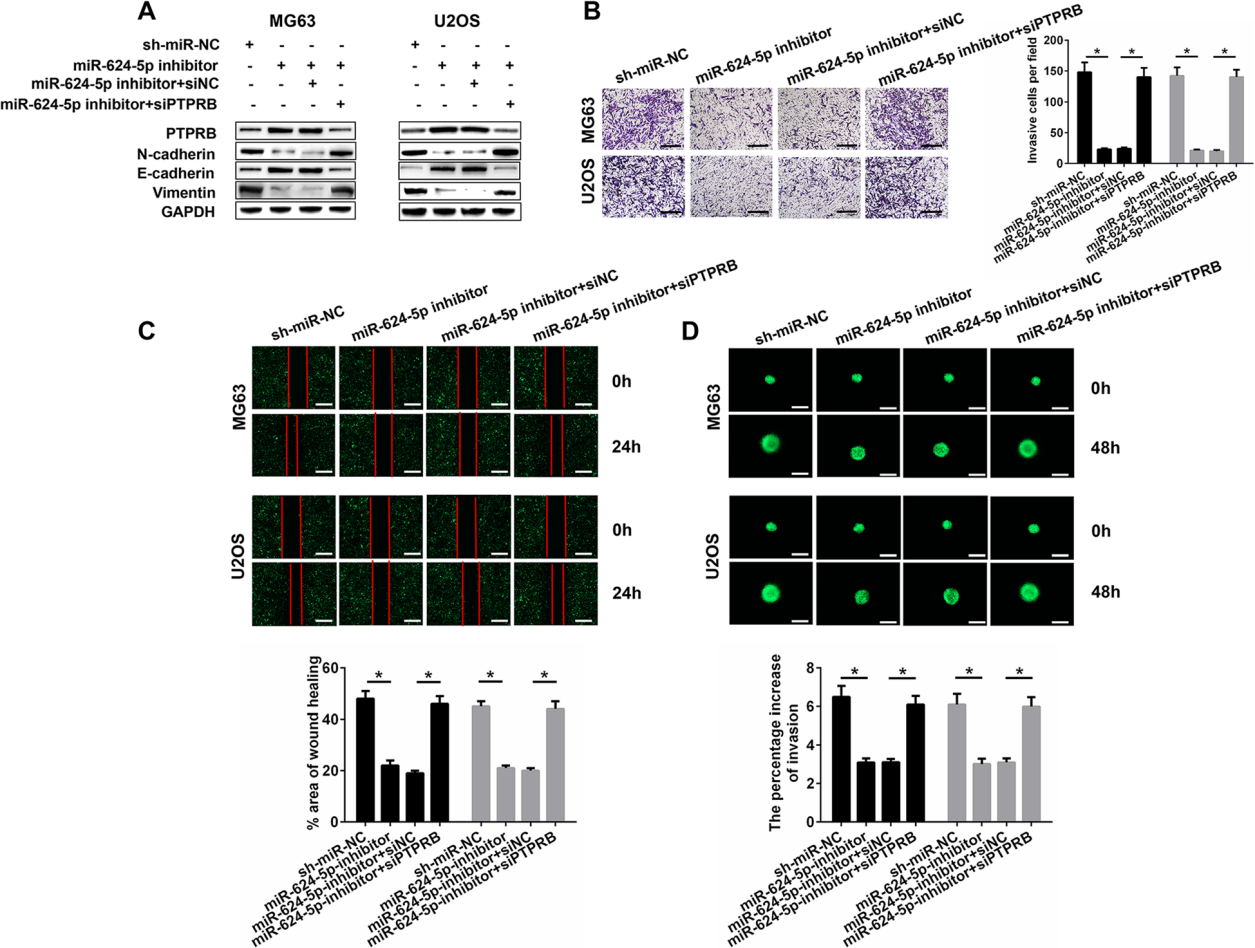
siPTPRB abolishes the effects of miR-624-5p inhibitor on OS cells in vitro. **a.** Western blotting shows that the effects of the miR-624-5p inhibitor on OS cells invasion and migration were significantly reversed by the application of siPTPRB (n = 3). **b–c.** Suppressing PTPRB reduced the protective effects of miR-624-5p inhibitor on OS cell invasion and migration (n = 4). **d.** Results of 3D spheroid BME cell invasion assays resembled the above consequences (n = 4)

To further verify that miR-624-5p regulates OS cell migration and invasion through targeting PTPRB, a series of rescue experiments were conducted in vitro. Firstly, Western blotting showed that the expressions of PTPRB and E-cadherin in MG63 and U2OS cells were notably increased by the inhibition of miR-624-5p, while the metastasis-related proteins such as N-cadherin and vimentin underwent a significant decline. Interestingly, these effects of miR-624-5p inhibitor were significantly reversed by the application of siPTPRB (Fig. 5a). The results of the Transwell assays suggested that suppressing PTPRB reduced the protective effects of OS cell invasion resulting from miR-624-5p inhibitor (MG63, *P* = 0.000; U2OS, *P* = 0.000; Fig. 5b). Similar rescue effects were found in MG63 and U2OS cells: the inhibitory influences of cell migration caused by miR-624-5p silencing in the wound-healing assays were abolished by PTPRB downregulation (MG63, *P* = 0.000; U2OS, *P* = 0.000; Fig. 5c). These results were also confirmed in the 3D spheroid BME cell invasion assays (MG63, *P* = 0.000; U2OS, *P* = 0.000; Fig. 5d).

### Overexpression of PTPRB restores the effects of miR-624-5p mimics on OS migration and invasion

**Fig. 6:**
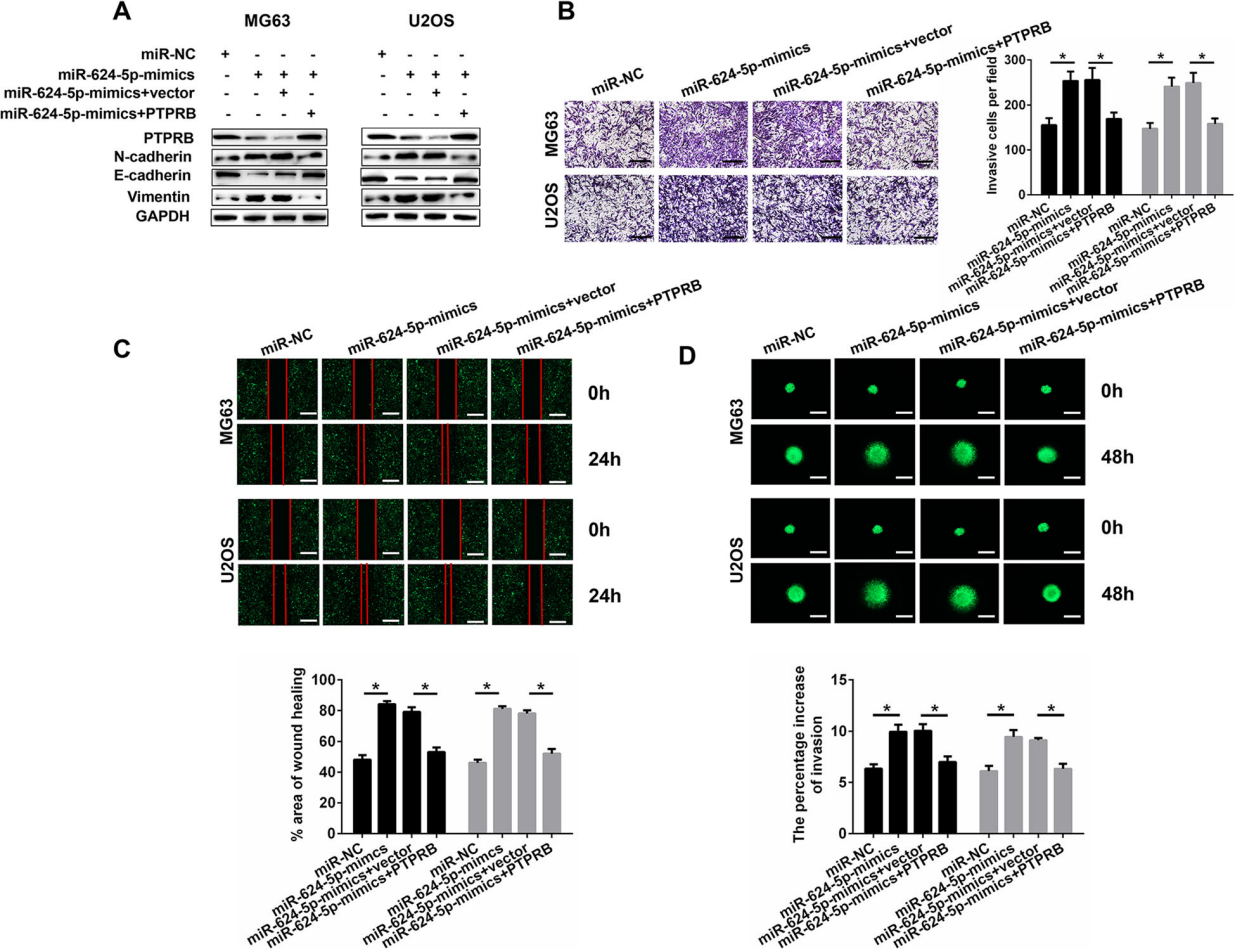
Overexpressed PTPRB restores the effects of miR-624-5p mimics on OS cells in vitro. **a.** Western blotting shows that OS cells invasion and migration was remedied by overexpressed PTPRB (n = 3). **b–c.** PTPRB could reverse the augmented OS cell invasion and migration caused by miR-624-5p mimics (n = 4). **d.** These above results were confirmed by 3D spheroid BME cell invasion assays (n = 4)

After we verified that the effects caused by miR-624-5p inhibitor on OS cell migration and invasion could be reversed by siPTPRB, a series of rescue experiments were conducted. We transfected OS cells with miR-624-5p mimics to realize miR-624-5p overexpression. Then, using Western blotting, significantly decreased expressions of PTPRB and E-cadherin in MG63 and U2OS cells were observed; on the contrary, N-cadherin and vimentin were remarkably enhanced. This time, these differences in protein expression were remedied by overexpressed PTPRB (Fig. 6a). The results of the Transwell assays also indicate that PTPRB could reverse the augmented OS cell invasion caused by miR-624-5p mimics (MG63, *P* = 0.000; U2OS, *P* = 0.000; Fig. 6b). Overexpression of PTPRB also reversed the effects of miR-624-5p mimics on OS cell migration according to the wound-healing assay results (MG63, *P* = 0.000; U2OS, *P* = 0.000; Fig. 6c). Of note, 3D spheroid BME cell invasion assays also confirmed the above results (MG63, *P* = 0.000; U2OS, *P* = 0.000; Fig. 6d).

To sum up, this part of our study demonstrated that knocking down PTPRB could abrogate the positive function of miR-624-5p inhibitor in OS migration and invasion. Contrarily, PTPRB was demonstrated to be helpful in suppressing OS cell migration and invasion resulting from miR-624-5p.

### miR-624-5p regulates the YAP/hippo signaling pathway through PTPRB

**Fig. 7:**
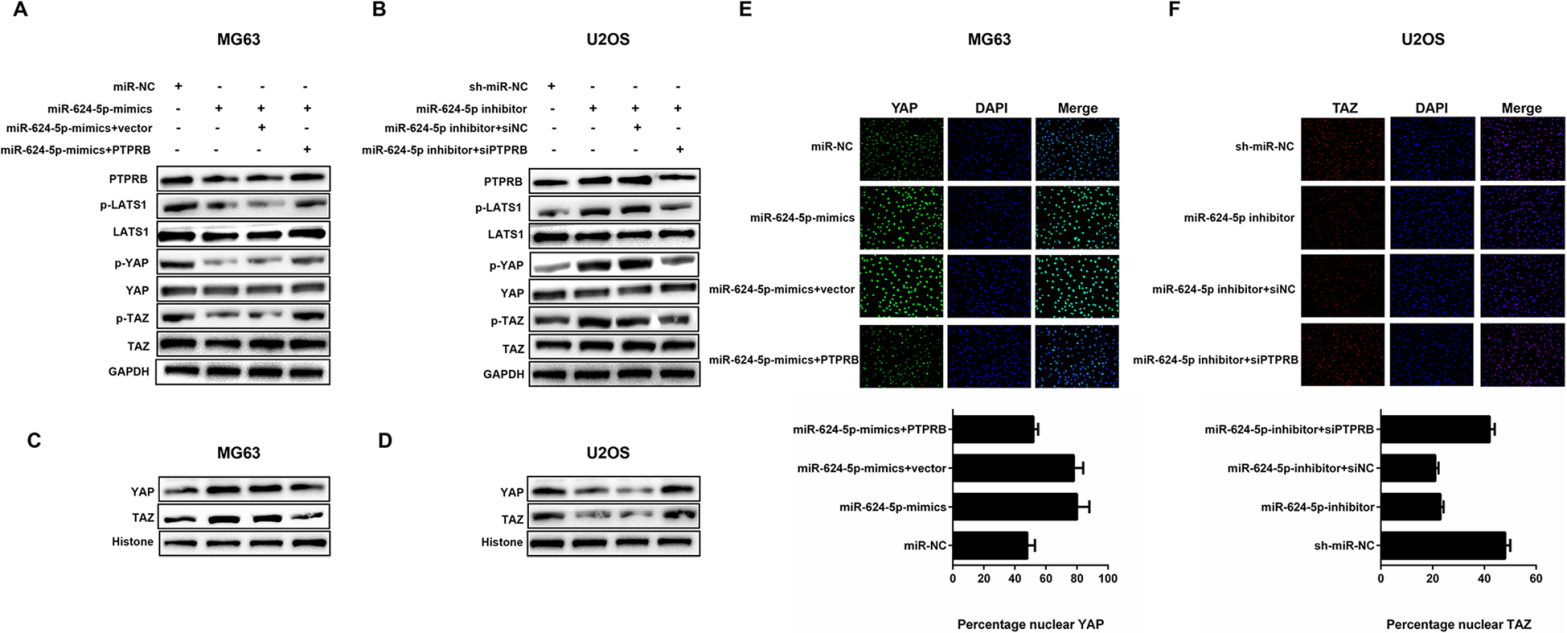
miR-624-5p regulates the YAP-Hippo signaling pathway through PTPRB. **a.** miR-624-5p mimics decreased the levels of PTPRB together with phosphorylation of LATS1 (p-LATS1), YAP (p-YAP), and TAZ (p-TAZ), but the suppressing effects were all remedied by overexpressed PTPRB (n = 3). **b.** Knockdown of miR-624-5p resulted in increased levels of PTPRB, p-LATS1, p-YAP, and p-TAZ, and the upregulation went into reverse when PTPRB was downregulated (n = 3). **c–d.** The levels of YAP and TAZ protein contained in nuclei were positively correlated with the miR-624-5p expression and had a negative correlation with the expression level of PTPRB (n = 3). **e.** miR-624-5p mimics enhanced YAP import into the nuclei of MG63 cells, and PTPRB could abolish this effect (n = 5). **f.** Less TAZ protein was transported into U2OS nuclei as a result of miR-624-5p suppression and the same restoration occurred when PTPRB was silenced (n = 5)

Western blotting and immunofluorescence analysis were conducted to investigate the underlying mechanism of how the miR-624-5p/PTPRB axis modulates OS cell migration and invasion. The Hippo signaling pathway has been reported to be involved in the development of a variety of cancers. All these studies suggested that it exerted a tumor-suppressing function in a broad range of tissues, including the muscular and skeletal systems [27–29]. Hence, we were interested in whether miR-624-5p and PTPRB affected OS invasion and migration through the Hippo pathway. Western blotting showed that miR-624-5p mimics in MG63 cells decreased the level of PTPRB, as well as downregulated the expression levels of phosphorylated LATS1 (p-LATS1), YAP (p-YAP), and TAZ (p-TAZ); however, the suppressing effects were all remedied by overexpressing PTPRB (Fig. 7a). On the other hand, the knockdown of miR-624-5p in U2OS cells resulted in increased levels of PTPRB, p-LATS1, p-YAP, and p-TAZ. Similarly, the upregulation went into reverse when PTPRB was downregulated (Fig. 7b). Furthermore, we observed that the levels of YAP and TAZ proteins contained in the nuclei were positively correlated with the miR-624-5p expression and had a negative correlation with the expression level of PTPRB (Fig. 7c, d). Our immunofluorescence analysis provided vigorous evidence that miR-624-5p mimics enhanced YAP import into the nuclei of MG63 cells and that PTPRB could abrogate this effect (*P* = 0.000, Fig. 7e). Conversely, less TAZ proteins were transported into U2OS nuclei as a result of miR-624-5p suppression, and the same restoration occurred when PTPRB was silenced (*P* = 0.000, Fig. 7f). These abovementioned results indicate that the miR-624-5p/PTPRB axis regulates OS cell migration, invasion, and metastasis via the YAP/Hippo signaling pathway.

### miR-624-5p accelerates xenograft tumor growth in vivo

**Fig. 8:**
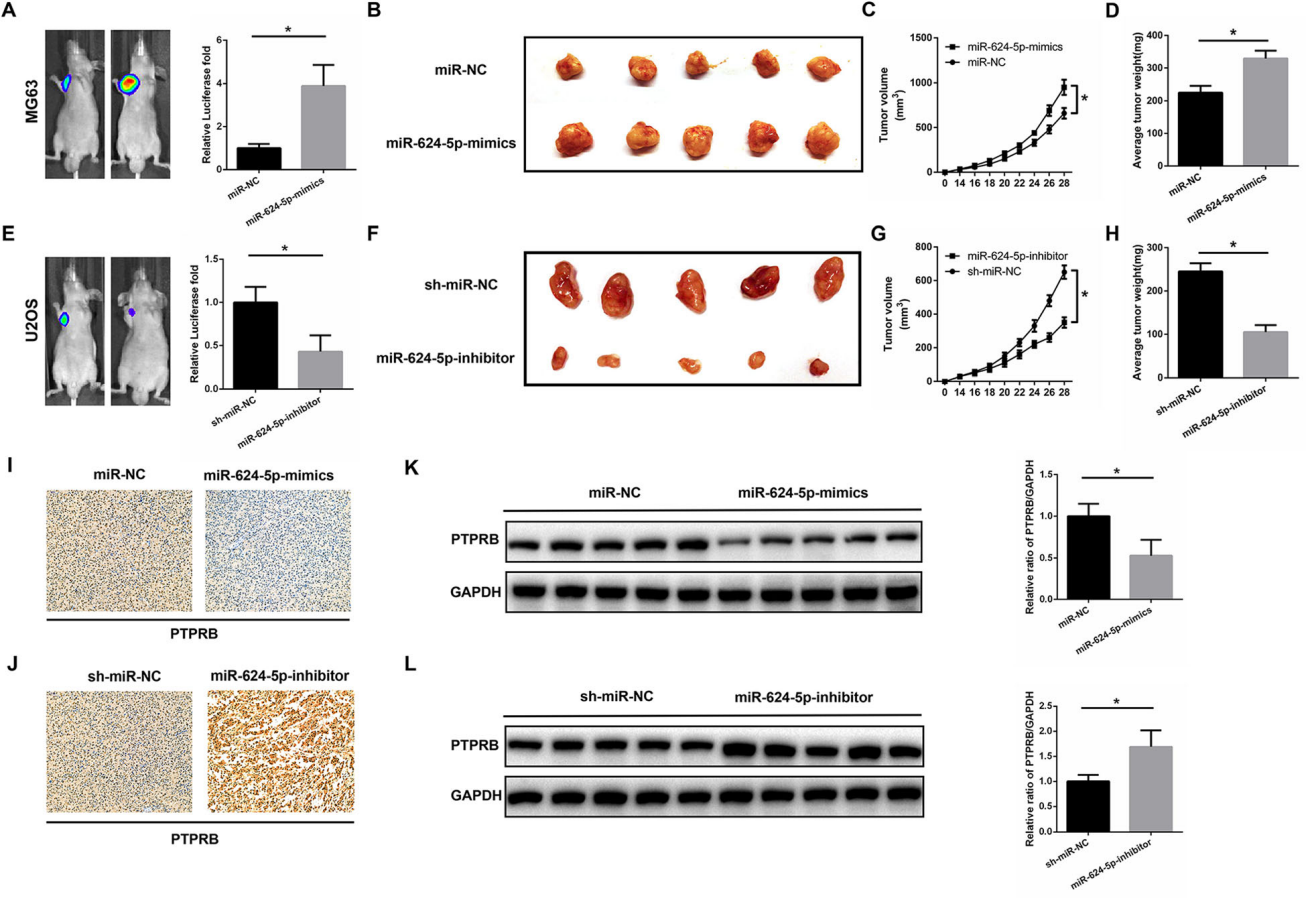
miR-624-5p accelerates xenograft tumor growth and pulmonary metastasis in vivo. **a–d.** MG63 cells with high miR-624-5p expression markedly promoted tumor growth in nude mice. The tumor volume was larger and the average tumor weight was heavier in the miR-624-5p mimics group (n = 5). **e–h.** Downregulation of miR-624-5p inhibited the tumor growth of U2OS cells, and tumor volume and average tumor weight were smaller and lighter, respectively, than the sh-miR-NC group (n = 5). **i–j.** The immunohistochemistry results show that PTPRB expression was reduced in the miR-624-5p mimics group and enhanced PTPRB expression was observed in the miR-624-5p inhibitor group. **k–l.** Western blotting results showed that PTPRB expression was reduced in the miR-624-5p mimics group and enhanced in the miR-624-5p inhibitor group (n = 5)

MG63 cells stably overexpressed or U2OS cells with downregulated miR-624-5p were subcutaneously injected into nude mice to examine the effects of miR-624-5p on tumor growth in vivo. Cells transfected with miR-NC or sh-miR-NC were used to treat nude mice as negative controls. The tumor sizes were monitored every 2 days from 14 days after injection, and the mice were euthanized after 4 weeks. Compared with controls, MG63 cells with high miR-624-5p expression markedly promoted tumor growth in nude mice (*P* = 0.000, Fig. 8a, b). The tumor volume was larger and the average tumor weight was heavier in the miR-624-5p mimics group (*P* = 0.000, Fig. 8c; *P* = 0.003 Fig. 8d). In contrast, downregulation of miR-624-5p inhibited the tumor growth of U2OS cells (*P* = 0.001, Fig. 8e, f), and the tumor volume and average tumor weight were smaller and lighter, respectively, than in the sh-miR-NC group (*P* = 0.000, Fig. 8g; *P* = 0.000, Fig. 8h). Next, we conducted immunohistochemistry to elucidate PTPRB expression in xenografts. The results showed that PTPRB expression was lessened in the miR-624-5p mimics group and enhanced PTPRB expression was observed in the miR-624-5p-inhibitor group (Fig. 8i, j). The Western blotting results also supported the abovementioned PTPRB expression variation in xenografts (*P* = 0.003, Fig. 8k; *P* = 0.002, Fig. 8l). In short, we demonstrated that miR-624-5p plays an important role in enhancing OS tumor growth in vivo.

## Discussion

As introduced above, OS is the most common primary malignant bone sarcoma in children and adolescents [30]. miRNAs are small noncoding RNAs that post-transcriptionally regulate gene expression. Previous studies have revealed the abnormal miRNA expression as an important regulator in the progression of OS [12, 31–33]. Thus, to illuminate the relationship between miR-624-5p and OS, in this current study, we conducted a series of experiments and revealed that miR-624-5p was upregulated in OS tissues and several cell lines. In addition, overexpressed miR-624-5p correlated with higher malignancy of OS, including enhanced cell invasion, larger tumor size, and higher metastasis potential. These data indicate that miR-624-5p is involved in OS development and could serve as a novel target for OS treatment.

Although the roles of numerous miRNAs in malignant tumors have been reported, little research has revealed the function of miR-624-5p on tumor progression. miR-624-5p was proposed in a recent study as an effective tumor suppressor via inhibition of the Wnt pathway only [16]. However, we found via real-time quantitative PCR that the expression of miR-624-5p was higher in OS tissues and cell lines than in the peritumor samples and normal cells. Interestingly, here, miR-624-5p did not present an antitumoral effect, which may prompt us to consider that miR-624-5p plays distinct biological roles according to different tumor types. Based on the discovery of aberrantly high expression of miR-624-5p, we explored the biological functions of miR-624-5p in OS progression as it was still unknown.

EMT is strongly associated with cancer progression and metastasis. A typical characteristic of EMT is the molecular changes of the epithelial cancer cells, which accelerate the epithelial feature loss and the acquisition of mesenchymal qualities. It is these central features that enhance cancer cell migration and invasion and antitumoral therapy resistance [34]. Additionally, other characteristics of EMT include downregulated E-cadherin and upregulated N-cadherin and vimentin levels [35]. In our study, a decreased E-cadherin level was found when miR-624-5p was overexpressed. Contrarily, increased levels of vimentin and N-cadherin prompted that tumor progression and metastasis was enhanced by an upregulated miR-624-5p level. The promoted ability of OS cell metastasis was also in accordance with our in vitro Transwell and wound-healing assays, which confirmed miR-624-5p as a risk factor in OS progression. Besides the risk role of miR-624-5p in OS cell proliferation, migration and invasion in vitro and in vivo, accumulating evidence have shown that PTPRB is one of the target genes of miR-624-5p. Firstly, miR-624-5p suppressed PTPTB expression in OS cells at both the mRNA and protein levels. Secondly, the luciferase activity of WT-PTPRB-3′-UTR but not MUT-PTPRB-3′-UTR was restrained by miR-624-5p. Thirdly, miR-624-5p was negatively correlated with PTPRB levels in OS tissues. PTPRB was downregulated in OS cells and recovery of its expression could abolish the effects of miR-624-5p. PTPRB, a type of protein tyrosine phosphatase receptor (PTPR), is also known as a kind of vascular endothelial protein tyrosine phosphatase. PTPRs were previously regarded as tumoral suppressors and are inactivated due to genetic mutations in human cancer [36, 37]. PTPRB was reported to be downregulated in non-small cell lung cancer tissues and serves as an independent biomarker for a patient’s prognosis [38]. As a negative regulator of vascular growth factor tyrosine kinases, PTPRB was found to harbor predominantly truncated mutations in 26% of tumor patients [26]. It was also found that higher expressions of PTPRB predict favorable survival rates among pulmonary carcinoid tumor patients. Considering that we have confirmed that PTPRB is markedly downregulated in OS tissues compared to adjacent tissues, these findings indicate that PTPRB has a suppressive role in OS tumorigenesis and progression.

The Hippo signaling pathway is critical for organ size control and tissue overgrowth management. When its activity is attenuated, pathological phenotypes, such as cancer, are prone to arise [22, 39]. The Hippo signaling pathway was identified by the Cancer Genomic Atlas as one of the eight major pathways that are commonly altered in human cancers [40]. Aberrant function of the Hippo pathway has been considered to take part in various cancer types, such as neurofibromatosis, mesothelioma, renal cell carcinoma, cervical squamous cell carcinoma, and basal cell carcinoma, etc. [41–45]. The core mechanism of the Hippo pathway is that it suppresses YAP transcription by activating LATS kinases, which causes YAP phosphorylation and degradation. Hence, we further tested whether miR-624-5p inhibits the Hippo pathway through PTPRB. Western blotting showed that overexpression of miR-624-5p decreased the levels of p-LATS1, p-YAP, and p-TAZ together with the downregulated PTPRB. Conversely, downregulating miR-624-5p led to increased levels of the above proteins in OS cells. What is more, the application of PTPRB or siPTPRB significantly remedied the effects of miR-624-5p mimics or inhibitors on the Hippo signaling pathway. Immunofluorescence further revealed that more YAP and TAZ proteins were transported to the nuclei of OS cells while the overexpression of miR-624-5p and PTPRB abolished this effect.

## Conclusions

In this study, we found miR-624-5p to have a promoter role for OS development. We showed that miR-624-5p accelerated tumor progression both in vitro and in vivo. We further confirmed that miR-624-5p inhibited Hippo signaling activity by directly suppressing PTPRB. However, the specific mechanism of PTPRB affecting the Hippo pathway remains unexplored and requires further investigation. In conclusion, we demonstrated the tumor-promoting role of miR-624-5p in osteosarcoma progression and its underlying mechanism through PTPRB and the Hippo pathway. In our opinion, this study revealed the important role of miR-624-5p and Hippo signaling pathway in the OS development. It may provide further insight into the progression of osteosarcoma. Thus, it is a reasonable inference that miR-624-5p antagonism may exert therapeutic values in OS, which may control the OS proliferation and metastasis, and even improve survival rate and life quality for the patients in the future.

## Supplementary information

**Additional file 1: Fig. S1.** miR-624-5p had the potential ability to promote OS cell proliferation. **A–B**. The Cell Counting Kit-8 (CCK-8) assay and colony formation analysis reveal that downregulating miR-624-5p significantly reduced cell proliferation after 5 days (**A**, *n* = 4; **B**, n = 4). **C–D.** Upregulation of miR-624-5p significantly increased proliferation of the MG63 and U2OS cells (**C**, n = 4; **D**, n = 4).

## Data Availability

Most of the datasets supporting the conclusions of this article are included within this article and the additional files. The datasets used or analyzed during the current study are available on reasonable request.

## Abbreviations

EMT: Epithelial-mesenchymal transition
IHC: Immunohistochemistry
OS: Osteosarcoma
PTPRB: Protein tyrosine phosphatase receptor type B
TAZ: Tafazzin
YAP: Yes-associated protein
