# Supplementary figures and images for: miR-624-5p promoted tumorigenesis and metastasis by suppressing hippo signaling through targeting PTPRB in osteosarcoma cells
Source: J Exp Clin Cancer Res. 2019 Dec 11;38:488. doi: 10.1186/s13046-019-1491-6 (PMC6907337; doi:10.1186/s13046-019-1491-6)

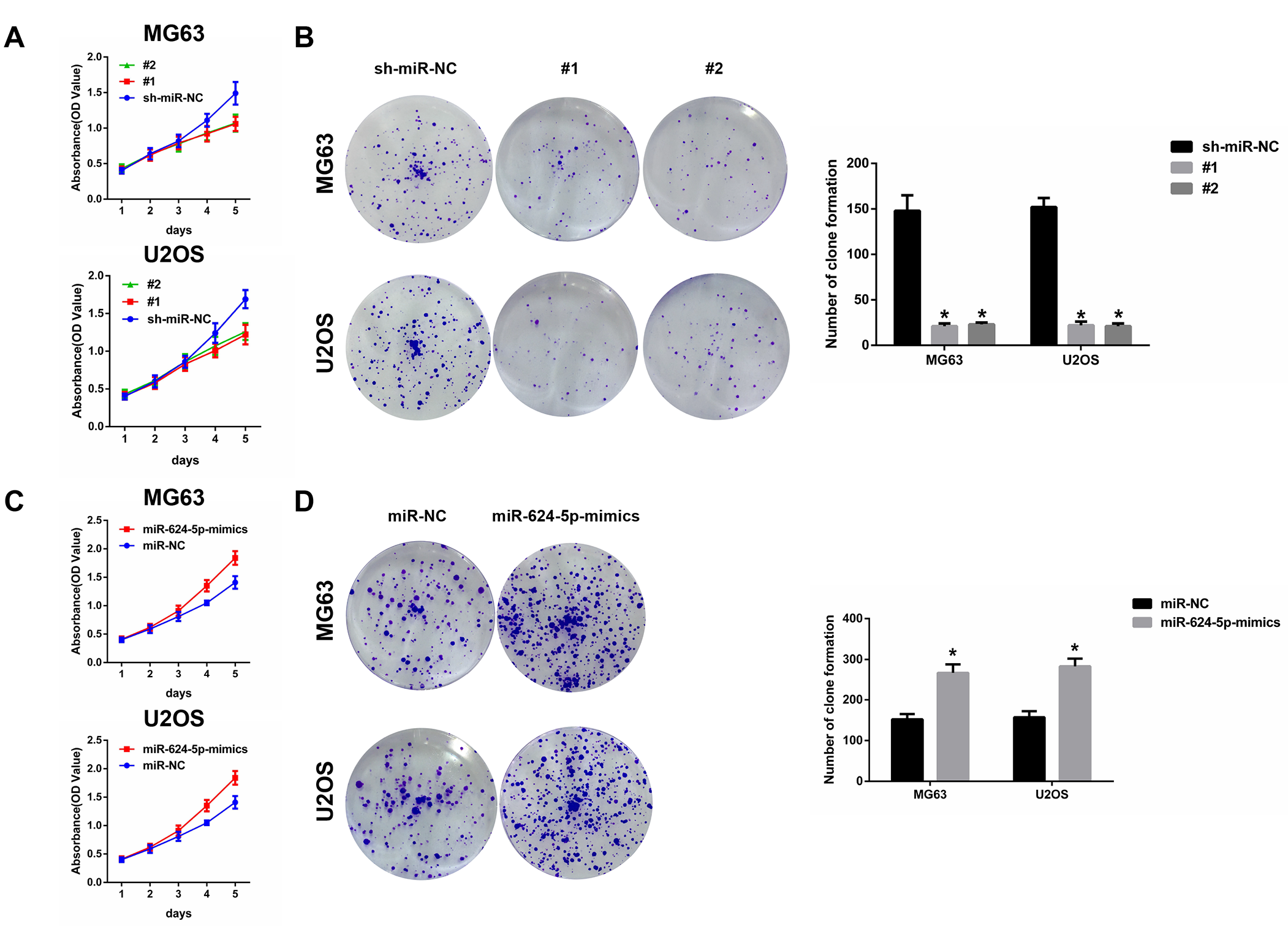

Supplement: Supplementary file 1 — Additional file 1: Fig. S1. miR-624-5p had the potential ability to promote OS cell proliferation. A–B. The Cell Counting Kit-8 (CCK-8) assay and colony formation analysis reveal that downregulating miR-624-5p significantly reduced cell proliferation after 5 days (A, n = 4; B, n = 4). C–D. Upregulation of miR-624-5p significantly increased proliferation of the MG63 and U2OS cells (C, n = 4; D, n = 4). [file 13046_2019_1491_MOESM1_ESM.tif]
